# Identification of hybrid node and link communities in complex networks

**DOI:** 10.1038/srep08638

**Published:** 2015-03-02

**Authors:** Dongxiao He, Di Jin, Zheng Chen, Weixiong Zhang

**Affiliations:** 1 School of Computer Science and Technology, Tianjin University, Tianjin. 300072, P. R. China; 2 Department of Computer Science and Engineering, Washington University, St. Louis. MO 63130, USA; 3 Institute for Systems Biology, Jianghan University, Wuhan. Hubei 430056, P. R. China; 4 Department of Genetics, Washington University, St. Louis. MO 63130, USA

## Abstract

Identifying communities in complex networks is an effective means for analyzing complex systems, with applications in diverse areas such as social science, engineering, biology and medicine. Finding communities of nodes and finding communities of links are two popular schemes for network analysis. These schemes, however, have inherent drawbacks and are inadequate to capture complex organizational structures in real networks. We introduce a new scheme and an effective approach for identifying complex mixture structures of node and link communities, called hybrid node-link communities. A central piece of our approach is a probabilistic model that accommodates node, link and hybrid node-link communities. Our extensive experiments on various real-world networks, including a large protein-protein interaction network and a large network of semantically associated words, illustrated that the scheme for hybrid communities is superior in revealing network characteristics. Moreover, the new approach outperformed the existing methods for finding node or link communities separately.

Most complex systems in various fields, such as social networks in social science, the Internet in engineering, and signaling pathways in biology, can be formulated as networks where nodes represent entities (e.g., individuals in a social network) and links represent some relationship between nodes (e.g., co-worker relationship in a social network). Individual entities in a complex system seldom exist in isolation, but rather are often organized in groups to exert functions. For example, an organization typically consists of units of different but related functions that interconnect in particular structures to maximize the overall performance of the organization. In biology, a group of proteins in a cell interact to form an RNA polymerase for transcription of genes. Therefore, a critical step toward understanding complex systems is to uncover organizational or community structures in the networks^1^. Communities, also referred to as clusters or modules, are groups of nodes that share common properties or play similar roles^2^. A primary objective of community detection is to identify sets of nodes with common functions by using information of network topology.

Many methods for community identification have been proposed. The most popular ones belong to the scheme for detecting node community^1^,^2^,^3^,^4^,^5^,^6^,^7^,^8^, a.k.a., *node scheme*, where communities are subsets of nodes relatively densely connected within groups but sparsely connected across groups^4^. Indeed, many real networks carry structures that can form node communities^4^,^5^,^6^,^7^,^8^.

In the conventional node scheme, a node belongs to only one community. However, overlapping community structures are ubiquitous in real networks^9^. For example, an individual has a family and belongs to a group of co-workers, each of which has its own function and forms its own circle of influence. Forcing a node into one community will fail to accommodate multiple relationships and functions that a node may have, resulting in erroneous representation of the network structure^9^.

To overcome this drawback, the link-community scheme has been proposed^10^. In this *link scheme*, links with a similar relational property form communities so that a node can inherit the community memberships of its adjacent links and, as a result, can naturally belong to multiple communities. There are real-world systems that can be represented by link communities^10^,^11^,^12^,^13^,^14^.

However, the link scheme typically generates a highly overlapping community structure even though a network may not have overlapping structure at all^3^. Take the American college football network^4^ as an example, which is to be elaborated in the Results section. Under the link scheme, this network produced a highly overlapping community structure with 83 of the 115 nodes overlapped one another, despite that the football teams are organized in conferences that have no overlapping structure. This problem stems from the fact that the link scheme forces every link into a community while in reality there are cases where some (background) links may better not be put into any community. For example, in protein-protein interaction (PPI) networks, some constitutive interactions, e.g., interactions among the proteins that form the basal transcription machinery such as RNA polymerase II, may better be ignored in a PPI network for analyzing PPIs along an aberrant pathway underlying a particular disease.

Many real-world systems have complex structures that are better characterized by a mixture of node and link communities. This suggests that a hybrid node-link community scheme, or *hybrid scheme* for short, will be more effective and robust in revealing and representing complex organizational structures than either the node or link scheme. In the hybrid scheme, a network can be characterized by a number of communities, where a community can be either a node community or a link community but not both. A node in the network may belong to a node community or be connected by an edge associated with a link community. Likewise, an edge in the network may be in a link community or be connected to a node associated with a node community. An illustrative example, from the data compiled by Knuth, is a network of 77 characters and their joint appearance in common scenes in Hugo's classic novel *Les Misérables*^15^, where nodes are characters and two nodes are connected if the two characters appear together in a scene. The node and link schemes can produce distinctive community structures (Fig. 1A and 1B). Since a node is forced into one community in the node scheme, multiple community memberships are lost under this scheme. For example, Fantine is classified only into the pink community (the pink node in box to the left of Fig. 1A). In fact, Fantine and the seven blue nodes form another community (the clique of the seven blue nodes plus the pink node for Fantine in Fig. 1A), which is a small social group consisting of four Parisian students and their respective lovers. Therefore, the node scheme misses this important relationship between Fantine and the group which she actually belongs to because it cannot properly characterize nodes with more than one role. This issue is exacerbated for the protagonist Valjean and his nemesis Javert (the other two pink nodes in box in Fig. 1A) who play more social roles than Fantine does and connect to ~48% of all the characters. The link scheme, on the other hand, may avoid such problem by allowing nodes to exist in more than one community. However, it has its own drawbacks. For example, the link between Valjean and Bossuet is placed into the pink link community (the pink link connecting the two nodes in box in Fig. 1B) so that Bossuet is forced into this community. However, Bossuet does not appear together with the members of the pink community in any of the scenes except Valjean who belongs to not only the pink community but also four other communities. Thus, it is problematic to place Bossuet into the pink link community. A similar problem occurs with the link between Fantine and Thenardier as the latter does not appear together with the members of the pink community except Fantine.

In sharp contrast, the hybrid node-link scheme can provide elegant solutions to these problems and correctly place multi-role characters into the right communities (Fig. 1C). In the hybrid scheme, a node may or may not be assigned to a node community and a link may be involved in a link community or set for free, depending on the objective for forming communities. In the *Les Misérables* example, Fantine was put into both the blue link community and the pink node community, and Valjean and Javert were also correctly assigned to multiple communities, thereby fixing the problem of the node scheme. Moreover, the hybrid scheme did not force the link between Valjean and Bossuet and the link between Fantine and Thenardier into any community so that Bossuet (and Thenardier) was free from the pink community, fixing the problem of the link scheme.

However, it is challenging to detect hybrid node-link communities, which requires to accurately characterize such structures. A viable approach is stochastic modeling which, instead of directly detecting communities, describes how such structures are generated in the first place. In this paper, we introduce a probabilistic model to accommodate both node and link communities, where we describe each community as a random graph that does not have any community structure and cannot be further subdivided. We develop two methods – an expectation-maximization (EM) algorithm and a nonnegative matrix factorization (NMF) approach – to estimate the probability that a node or an edge belongs to a node or link community. Based on the learned model parameters, we adopt a heuristic approach to infer the hybrid node-link community structure that best characterizes the observed network. We call the proposed method NLC (Node-Link Communities), which can be run to find node, link or hybrid node-link communities as so desired.

## Results

We performed three experiments. The first was to demonstrate the favorable features of the new scheme for hybrid communities over the existing schemes for single type of communities. The second was to show the superior performance of our NLC method over the existing methods for finding a single type of communities (i.e., node or link communities). The third was to apply NLC under the hybrid scheme to two applications in biology science and cognitive psychology, where several rich metadata can be used as gold standards for validation, to show the superior performance of NLC over the existing methods in solving real-world problems. Here our NLC method appears in two versions: NLC-EM and NLC-NMF, which correspond to the EM algorithm and the NMF approach, respectively.

Since our model takes the number of communities *c* as a parameter, for the first two experiments, we used the generalized map equation^16^ (see Methods) to search for the target community structure by iterating over possible values of *c*. The generalized map equation is based on the principle of minimum description length (MDL)^17^ and as such is particularly suitable for overlapping communities. Under this measure, the shorter the MDL of an overall community structure, the better the structure is. As the two networks considered in the applications are very large, it is not practical to determine the number of communities *c* by searching for the best structure among all candidates with different *c*. To address this issue, we adopted an iterative bipartition strategy to determine the number of communities *c* for large networks in the applications.

In order to evaluate and compare the different results, a suitable metric for the goodness of a community structure is required. Most of the current quality measures are designed for non-overlapping structures. When extended, these methods penalize overlapping structures^18^. Fortunately, the generalized map equation^16^ based on MDL^17^ can naturally measure overlapping communities. Here we used this quality metric to evaluate community structures from different methods when the actual network community structures were unknown. On network structures with the true community structures available, we adopted the normalized mutual information (NMI)^19^, a widely used accuracy metric, for evaluating network methods. Moreover, as the two large networks in the applications possess rich metadata, we evaluated the performance of different methods by measuring how well the discovered community structures reflect the metadata, which appeared to be more convincing than using quality metrics designed only based on network topology.

### Comparison of the three community schemes

The NLC method supports the three schemes for the identification of hybrid community structures as well as node and link communities separately. We thus applied it to identify the best network structures for each of these schemes. The comparison was done on three real network problems. For simplicity, here we present the results of NLC-EM.

#### Zachary's karate club

The Zachary's “karate club” network^20^ has become a *de facto* testbed for community detection algorithms. Fig. 2 shows the community structures from the three schemes. Three disjoint node communities (Fig. 2A) were identified in the node scheme. Node 1 (the instructor, the red node in box) was exclusively assigned to the red community, even though it is also connected to all the nodes (except one) in the purple community, showing its importance to the purple community. In comparison, the hybrid (and link) scheme (Fig. 2B) correctly placed node 1 (square node in box) in the purple and red link communities. Moreover, the MDL for the communities in the hybrid (and link) scheme is 4.2966, which is smaller than that for node partition (4.3563).

To further evaluate the quality of the results from the three schemes, we compared the MDLs of the structures from these schemes with the community number *c* varied. As shown in Fig. 3, the results from the hybrid scheme have smaller MDLs than the other two schemes except when *c* equals to 3 at which the hybrid and link schemes produce the same network structure. The result in Fig. 3 also suggested that there should be 3 communities, whereas the reported “actual” number was two. In fact, the instructor's faction (square nodes) contains two evidently overlapping subgroups that were connected only through the instructor (node 1, Fig. 2B). Thus, it is more suitable to split the instructor's faction into two.

#### American college football network

In the American college football network^4^, the nodes represent football teams and a link represents a game played by two teams during the football season in year 2000. The teams were divided into “conferences”, which formed actual communities. The teams in the same conference played more often with the teams not in the same conference. A team played on average approximately 7 intra- and 4 inter-conference games in the season. This suggested that the network possessed typical characteristics of node communities. As expected, the hybrid scheme discovered a node-community structure for this network (Fig. 4A). In contrast, the link scheme produced a highly overlapping community structure with 83 out of all 115 nodes overlapped (Fig. 4B), revealing a serious drawback of this scheme. We compared the results from the hybrid and link schemes against the reported network structure using the extended normalized mutual information (NMI) for overlapping communities^19^. The hybrid scheme scored NMI = 0.8035 while the link scheme scored NMI = 0.3604, showing that the former significantly outperformed the latter. Furthermore, we also compared the community structures from the hybrid (node) scheme and the link scheme as well as the reported structure using the MDL quality metric. The MDL for the hybrid scheme (5.4487) was smaller than that for the link scheme (6.1125). Surprisingly, the MDL for the hybrid scheme was also smaller than that of the reported structure (5.6772). This may be due to two factors. First, the independent teams that did not belong to any conference were grouped into a separate but subjective “conference” in the reported community structure even though these independent teams did not play more often among themselves than with other teams. Second, our hybrid, data-driven community discovery scheme was able to more faithfully detect community structures underlying the data of overall games played than the reported result.

The MDL was used to evaluate the community structures obtained by the three schemes with varying number of communities. The detailed result is shown in Fig. 5. As this network has typical characteristics of node communities, the hybrid scheme always produced the same results as the node scheme, and the MDLs from the hybrid (and node) scheme are always smaller than that of the link scheme. The best network structure was found by the hybrid scheme with 12 node communities, which is the same as the actual number of conferences.

#### Les Misérables

**Figure 1:**
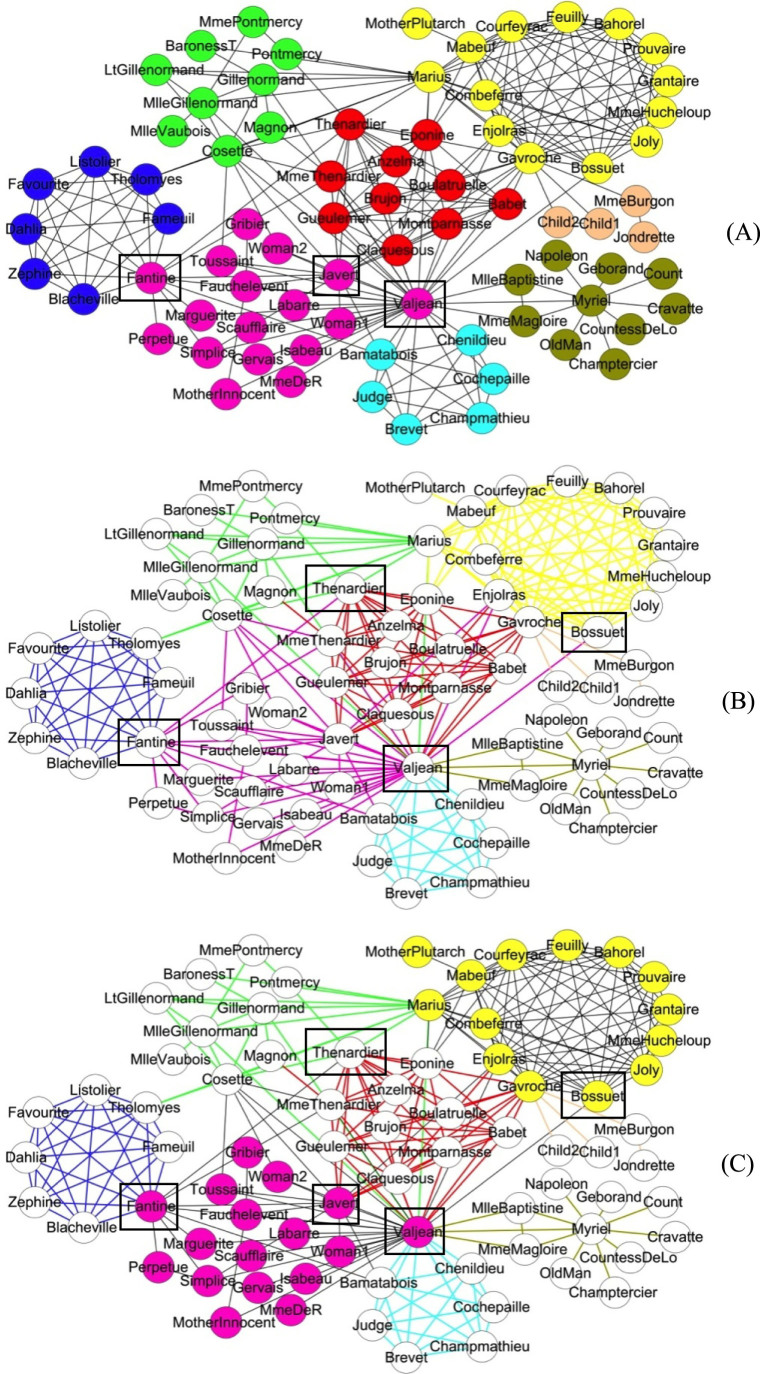
Community structures of the co-appearance network of characters in *Les Misérables* from (A) the node scheme, (B) the link scheme and (C) the hybrid node-link scheme. Here, node or link communities are colored in nodes or links respectively, and uncolored nodes and black links represent background.

**Figure 2:**
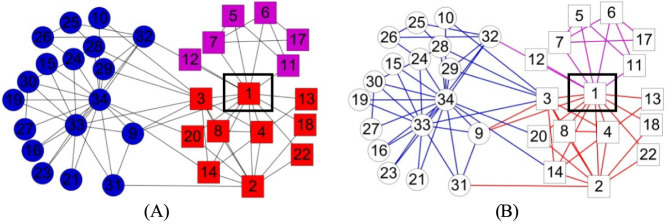
Communities of the “karate club” network obtained by (A) the node scheme and (B) the hybrid (and link) scheme. The nodes in circle and square represent the two communities as originally reported: the club administrator's faction in circles and the instructor's faction in squares. Node or link communities from our model are colored in nodes or links, respectively.

**Figure 3:**
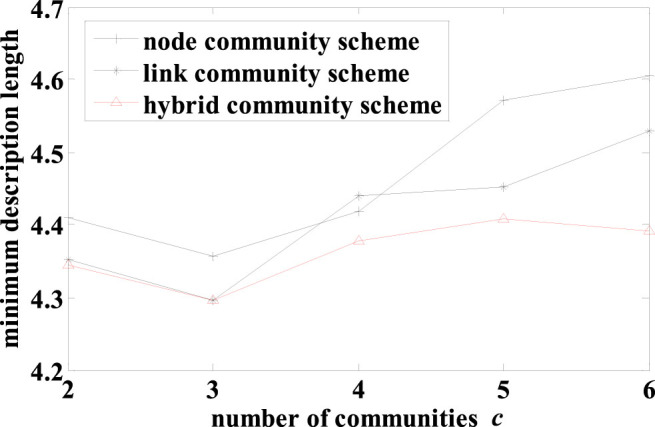
The minimum description lengths for the results from the three schemes with varying number of communities *c* on the “karate club” network. As shown, the hybrid scheme produced structures with the smallest MDLs and the best structure has three communities.

**Figure 4:**
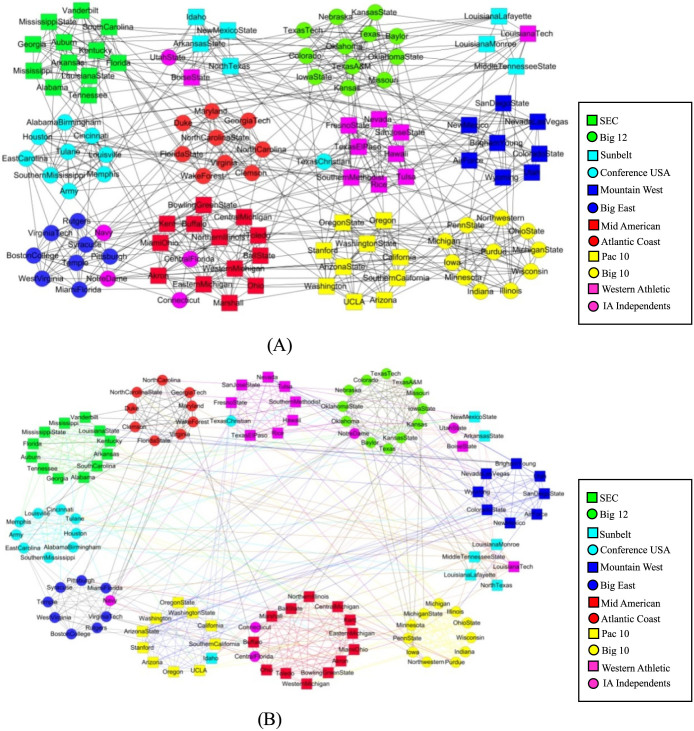
Community structures of American college football network obtained by (A) hybrid and node-community schemes and (B) the link-community scheme. Nodes in the network represent teams and links represent games between teams. Here, the 12 different combinations of node shape and node color represent the actual “conferences”. The clusters of nodes in space denote node communities obtained by our model in (A), and the colored links denote link communities from our mode in (B).

**Figure 5:**
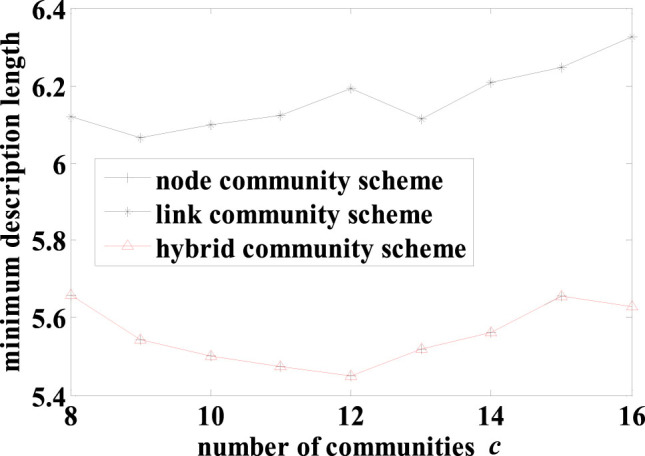
The minimum description lengths for results from the 3 schemes with varying number *c* of communities on the American college football network.

**Figure 6:**
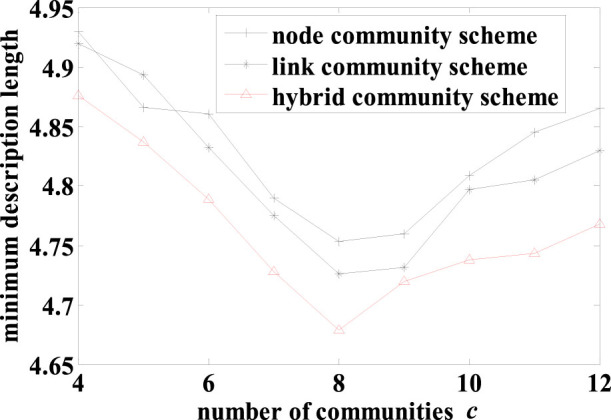
The minimum description lengths for results from the 3 schemes with varying number *c* of communities on *Les Misérables*.

The three distinct community structures for the three schemes are shown in Fig. 1. As discussed in the Introduction, the hybrid scheme can overcome the shortcomings of the node and link schemes. Furthermore, the MDL of the result from the hybrid scheme (4.6783) is less than that from the node scheme (4.7528) and link scheme (4.7259). Similar to the two early network problems, the results from the hybrid scheme on this co-appearance network have shorter MDLs than the node and link schemes with all values of community number *c* evaluated, which is shown in Fig. 6. The shortest description length was achieved with 8 communities (Fig. 1).

### Comparison with the existing methods

We evaluated the performance of NLC, including NLC-EM and NLC-NMF, along with several well-established methods for finding node communities or link communities on nine widely used real networks (Table 1). These methods included the Louvain method^6^ which is regarded as one of the best for node partitioning^1^, LC (Link Community)^10^ which is the most well-known method for link-community finding, and CPM (Clique Percolation Method)^9^ which is the most prominent algorithm for overlapping community detection. We also included two model-based methods proposed by Newman *et al*, i.e., NModel for node communities^21^ and LModel for link communities^13^.

**Figure 7:**
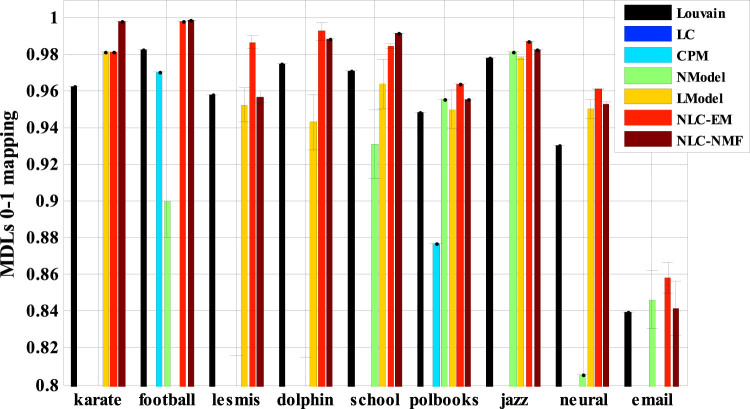
The 0–1 normalized MDLs of the results of the new NLC method (including NLC-EM and NLC-NMF) and five existing algorithms (in Table 1). For each network, we normalize the optimal MDL-value to 1 and its baseline MDL-value to 0; we then perform a linear normalization on its MDL-values for each of the compared methods.

As the ground truth of the community structures of these networks is not available, we used the MDL as the quality metric. As shown in Table 1, the new method NLC had the best performance on all networks. Specifically, NLC-EM had the best performance on 6 of the 9 networks, and NLC-NMF performed the best on the remaining 3 networks. The superior performance of NLC may be attributed to its flexibility and robustness in forming hybrid node-link communities. Further, we rescaled the MDL scores to better illustrate the improvement across different networks. In particular, for each network, we took its optimal MDL-value (calculated by Fuzzy Infomap^16^) as 1, and set its baseline MDL-value (averaged over 100 random partitions) to 0. The relative improvements are shown in Fig. 7. As shown, both NLC-EM and NLC-NMF outperformed each of the other 5 methods we compared.

Moreover, to further assess the performance of these methods, we compared them on six real networks with known community structures. These networks are originally constructed from the data of social media in the Stanford Network Analysis Project^22^, where the communities, including overlapping ones, in each of these networks are explicitly labeled. We thus evaluated these methods by comparing their predictions with the known, true communities. Note that this is an exact performance measure because the actual community structures, rather than some measure based on network topologies, were adopted in comparison. To serve our purpose, here we employed the widely used NMI index which has been extended to overlapping communities as the accuracy measure^19^.

The networks used here are very large (see Table 2), which exceed the capacities of almost all currently available community detection methods. We thus adopted a sampling method to obtain a large set of networks with manageable sizes. Similar to what was suggested by Yang & Leskovec^23^, we randomly selected a node *u* in the given graph *G* which belonged to at least two communities; we then took the subnetwork to be the induced subgraph of *G* consisting of all the nodes that shared at least one known community membership with *u*. Besides, in order to obtain credible subnetworks with well-defined overlapping community structures, for each network we combined duplicate communities and removed the communities containing no more than two nodes in their true structures; we then disregard the subnetworks whose values of extended modularity (EQ)^24^ under the ground-truth were less than a threshold of ε = 0.1, which could be considered as having no well-defined community structure. Finally, we generated 500 networks with overlapping communities for each of the 6 datasets in our experiments.

Quantified by NMI as the performance metric, our NLC method outperformed all the other methods on all six networks (Table 2). NLC-EM had the best performance on 2 of the 6 networks, and NLC-NMF performed the best on the remaining 4 networks. Besides, NLC-EM was on average 5.75%, 12.55%, 8.60%, 4.56% and 4.39% more accurate than Louvain, LC, CPM, NModel and LModel, respectively, and NLC-NMF was on average 11.17%, 17.96%, 14.02%, 9.98% and 9.81% more accurate than Louvain, LC, CPM, NModel and LModel, respectively. This result, which was independent of any network topologies like the MDL index uses, evidently illustrated the superior performance of NLC.

### Applications to large networks

We applied the hybrid scheme and the NLC algorithm to help elucidate the structures of a large protein-protein interaction network in biological science and reveal hidden associations among commonly used words. The domain specific results, such as protein-protein interactions and their biological implication, will be reported elsewhere. Here, we discuss the results on network structures identified using the domain metadata based function and in comparison with some existing methods.

In order to obtain an objective quality assessment beyond a network structural measure, such as MDL, we utilized domain knowledge specific to these applications to assess the quality of the results. Furthermore, the study was performed in comparison with three well-known methods (selected from Table 1) that can discover overlapping communities and are applicable to large networks. The methods compared included CPM^9^ which is the most prominent algorithm for detection of communities with overlapping structures, LC^10^ which is best known for link-community finding, and LModel^13^ which is the most related approach compared with our new method NLC. It is worth noting that CPM may not classify all nodes of a network into a community. It may treat some nodes of a network as background and not designate them to any community. To set a baseline for fair comparison, we designed two types of comparison. One was on the subgraph processed by CPM, and the other on the whole network. In the first comparison, the subgraph processed by CPM was taken as the targeted network. To be fair for all the methods, we used the number of communities attained by CPM as the number of communities for LModel and NLC in this comparison. As it is difficult to control the number of communities for LC^10^, we left LC to the second comparison. The second comparison was carried out on the whole networks using LC^10^, LModel and NLC. As the two networks considered here were much larger than those used before, it was not practical to determine the number of communities *c* by searching for the best structure among all candidates with different *c*. Thus, we adopted a simple partitioning strategy in NLC (and LModel) by repeatedly bipartitioning a (sub)network using our model (or LModel) until the likelihood function (or a loss function) could not be further improved. This strategy used NLC (and LModel) as a hierarchical clustering algorithm similar to the LC method, making the three methods more comparable for evaluation.

#### Protein-protein interaction network

The first large network considered was the protein-protein interaction (PPI) network of budding yeast *Saccharomyces cerevisiae*^25^. It contains 2,640 nodes (proteins) and 6,600 links representing physical interactions between pairs of proteins.

We used the terms in Gene Ontology (GO)^26^, the most elaborated gene function annotations, as domain metadata for quality assessment. The GO terms include information of functions and cellular locations of a gene and biological pathways that a gene may be involved in. The biological significance of a community of genes (nodes) can be measured by the GO terms enriched in the genes in the community, measured by the standard Fisher's Exact Test. In particular, the *p*-value of the test can be calculated using the probability of observing *k* proteins associated with a GO term, *t*, when randomly choosing *n* proteins from a pool of *N* proteins, where *m* of them are annotated with the term *t*. We further calculated the False Discovery Rate (FDR) adjusted for the Benjamini-Hochberg multiple testing correction^27^. A GO term was considered as significantly enriched in a community if its FDR was no greater than a specified threshold. To measure the biological significance of a community structure, we used as a quality metric the average number of significantly enriched GO terms with *p*-values not exceeding a threshold. The larger this average number of significant GO terms, the more biologically significant a community structure is.

**Figure 8:**
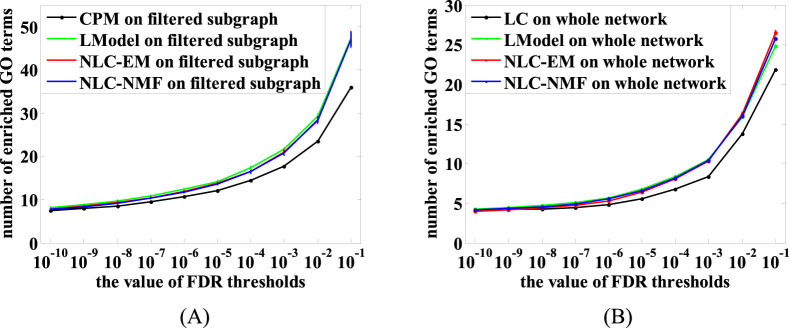
Comparison of NLC-EM, NLC-NMF, LModel and CPM (A) and NLC-EM, NLC-NMF, LModel and LC (B) on a large budding yeast PPI network in terms of the number of enriched GO terms that were statistically significant with FDR below a threshold. Error bars show the standard deviations from 50 runs, and CPM and LC are deterministic algorithms without standard deviations.

Our new methods NLC-EM and NLC-NMF identified PPI community structures with more significant GO terms than CPM under all 10 different FDR thresholds tested (Fig. 8A), showing their better performance over CPM. Note that NLC-EM and NLC-NMF ran on the subgraph that was processed by CPM, making the comparison biased in favor of CPM. Furthermore, NLC-EM and NLC-NMF outperformed LC as well using the same quality assessment (Fig. 8B). Besides, the performances of NLC-EM and NLC-NMF were competitive with that of the most related method LModel in both these two cases, shown in Fig. 8(A) and Fig. 8(B), respectively.

#### Word association network

The second large network deals with words and the associations among words that people typically intend to use. The network was constructed from the University of South Florida Free Association Norms data set^28^ and contained 5,017 nodes (words) and 29,148 links (association between pairs of words)^9^.

We used WordNet, which is an online lexical reference database^29^, as the domain metadata for quality assessment of community structures. In WordNet, words are organized in sets of cognitive synonyms, known as Synsets, each of which represents one lexical concept. In our analysis, we considered two words to be semantically related or similar when they belong to the same Synset. To assess the quality of a community structure, we computed the enrichment of similarity between a pair of nodes^10^
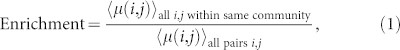
where *μ*(*i*,*j*) = 1, if words *i* and *j* belong to the same Synset, or 0, otherwise. In other words, the enrichment is the average similarity between all pairs of nodes that belong to a community, divided by the average similarity between all pairs of nodes. It quantifies how much a community structure differs from the baseline structure (the whole network) from the perspective of semantic similarity. The larger the enrichment, the better a community structure is.

**Figure 9:**
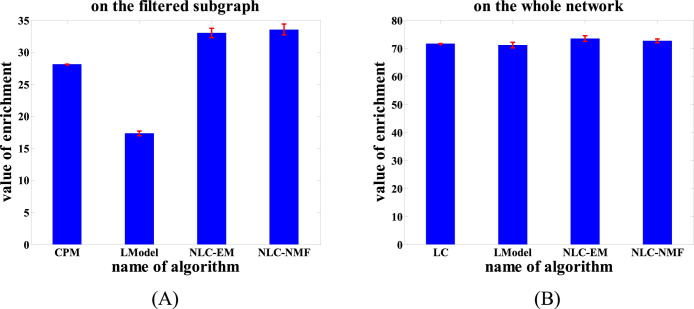
Comparison of NLC-EM, NLC-NMF, LModel and CPM (A) and NLC-EM, NLC-NMF, LModel and LC (B) on word association network in terms of the Enrichment defined by Eq. (1). Error bars show the standard deviations from 50 runs, and CPM and LC are deterministic algorithms without standard deviations.

We first compared NLC-EM, NLC-NMF, LModel and CPM, following the same comparison scheme as in the PPI network analysis. As shown in Fig. 9(A), the enrichments of the results from NLC-EM, NLC-NMF, LModel and CPM were 33.0076 ± 0.7332, 33.5391 ± 0.8724, 17.3054 ± 0.3515 and 28.0801, respectively, showing that NLC-EM (NLC-NMF) improved by 90.7% (93.8%) over LModel and by 17.5% (19.4%) over CPM, even though the comparison was in favor of CPM as the subgraph analyzed was chosen by CPM. In the second comparison against LModel and LC, the result from NLC-EM and NLC-NMF had enrichment values of 73.3797 ± 1.0431 and 72.5618 ± 0.6993, respectively, both of which were greater than that of 71.1465 ± 1.0173 from LModel and that of 71.5827 from LC (Fig. 9B). Note that the mean values and standard deviations of NLC and LModel were based on 50 trials, and CPM and LC are deterministic algorithms so that they do not have standard deviations. Therefore, NLC is more effective than the three popular existing methods in revealing semantic associations among words in the large word network.

## Discussion

While finding node and link communities together has been alluded to in early work, in this paper we presented a relatively thorough approach to developing methods for identifying hybrid node-link communities and analyzed its performance for characterizing complex network structures. An effective model and algorithm were developed to identify such hybrid communities. The hybrid scheme was able to overcome the inherent drawbacks of the node and link schemes, such as inability to support multiple roles that a node may play or forcing nodes to have unjustified relationships, which have limited the applicability of the node and link schemes. The analyses on several real networks, including a large protein-protein interaction network and a large word association network, demonstrated the superior performance of the hybrid scheme in revealing subtle and intricate network structures in real networks. The new NLC method, whose software is available from the authors, can be used separately to find node, link and hybrid node-link communities.

Stochastic models have been proposed, separately, for node communities^21^,^23^,^30^,^31^,^32^,^33^,^34^ and link communities^13^. However, they often fail to model the two types of communities together. Here, we developed a unified model of node and link communities. Different from the existing models that extend the classic stochastic blockmodel^35^, our new model generalizes the null model of modularity^5^ to incorporate the ability of describing mixed communities which the original null model does not possess. The centerpiece of the proposed model is an expected node degree function, which is optimized to fit the node degrees of a given network by using two different methods, i.e., an expectation-maximization algorithm and a nonnegative matrix factorization approach.

The most relevant previous work is the model proposed by Ball, Karrer & Newman^13^, which mainly focuses on the detection of link communities, and can be extended to node communities in principle. It may also be suitable for detecting node and link communities simultaneously. Although the Ball's model and our model presented here seemed to be similar, they have several key differences. First, the main purpose of our model is not only to accommodate the coexistence of node and link communities, but also to support the development of a hybrid node-link community scheme, which is our main contribution beyond the existing work for finding node or link communities separately. Second, although we used the idea of Ball's model that decomposes and combines probabilistic communities to describe networks, we employed a different way to describe each probabilistic community. Specifically, the Ball's model is parameterized by a set of parameters *θ_ik_*'s, where *θ_ik_* denotes the propensity of node *i* to have links in the *k*-th community; and then it takes *θ_ik_θ_jk_* as the expected number of links in the *k*-th community connecting nodes *i* and *j*, which is based on some statistically analysis. On the other hand, our model is parameterized by a set of parameters *d_ik_*'s, where *d_ik_* is defined as the expected node degree of *i* in the *k*-th community; and then it takes 
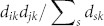
 as the expected number of links in the *k*-th community between nodes *i* and *j*, which is based on the widely accepted null model of modularity^5^. Third, in general one can map 
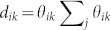
 from the Ball's model to our model. Then the Ball's model assigns a node to a community for which the value of 
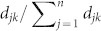
 is the largest. This means that one should first calculate the proportion of a node *i* with respect to all the nodes in each community *k* (
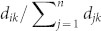
), and then select the community for node *i* in which the proportion is the largest. Roughly speaking, the community membership of node *i* is determined by its importance to the community compared with other nodes. In contrast, our model assigns a node to a community for which the value of 
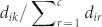
 is the largest, which means we select the community *k* for a node *i* to which this node devotes most of its contribution (
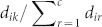
). Intuitively, in our model, node *i* chooses to join a community according to the resources that it devote to the community. Therefore, the node community structure derived from our model is different from that of the Ball's model. Fourth, our model has a constraint 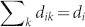 for each node *i*, i.e., the sum of the expected degrees of node *i* in all the communities equals to the actual degree of node *i* in the given network; while the Ball's model is not subject to such constraints. In the “Parameter learning based on expectation-maximization algorithm” section, this constraint can be automatically satisfied by using a Poisson distribution (Eqs. (8) and (9)), which results in the same EM algorithm as that proposed by Ball. But in the “Parameter learning based on nonnegative matrix factorization” section, when using a squared loss instead of a Poisson distribution to fit the model to the given network, the objective function of our model will have an effective constraint term (Eqs. (10) and (12)); while the Ball's model corresponds to 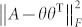 without this constraint. The extra constraint will make the solution space of these two objectives different and hence correspond to different community results. Finally, our model may be more easily extended to some newly appeared community detection variations. To be specific, by extending the null model, modularity *Q* has been applied in many new community detection problems, e.g., semi-supervised community detection^36^, dynamic community detection^37^, space-based community detection^38^, and community detection with structure and content^39^. Because we also use the null model as the base, one may easily use similar extension of the null model as did in these previous works to extend our model to these new problems.

Our NLC method can be regarded, in principle, as an approach for detecting overlapping communities of nodes. Recently, a number of approaches for overlapping community detection have been proposed. One of such approaches is based on the idea of clique percolation theory, i.e. that a cluster can be interpreted as the union of small, fully connected subgraphs that share nodes^9^,^24^,^40^. Another type of methods discovers each natural community that overlaps by using some local expansion or optimization approaches^19^,^41^,^42^. A third type of methods, namely link community detection, partitions links instead of nodes to discover community structures^10^,^11^,^12^,^13^,^14^; a node is considered to overlap with other nodes if the links connect to it belong to more than one cluster. Besides, many model-based methods^23^,^30^,^31^,^32^,^33^,^34^, which maintain probabilistic community membership of nodes, can also be extended to find overlapping communities. Of particular interest is the model proposed by Yang & Leskovec^23^,^34^. To better model overlapping communities, they remove the constraint that the sum of probabilities for each node belonging to different communities must be one, and describe networks with *dense community overlaps* which have been observed in real datasets but not been considered by other models. However, this type of methods often requires a threshold for the probabilistic memberships so as to get a community structure, which is difficult to determine for real applications^18^. Departure from the existing methods, our NLC method is for finding hybrid node-link community. Compared with other partitioning schemes, such as node community methods that focus mainly on nonoverlapping communities and link community methods that typically produce highly overlapping communities, the new hybrid scheme produces community structures with varying degrees of overlaps, and hence can better describe the true community structures of complex networks. Besides, the hybrid node-link community detection can be also taken as a type of new methods to find overlapping communities of nodes in networks.

A practical issue in network structure analysis is the lack of information of the number of communities to be targeted for. Neither a robust criterion nor an efficient method for this problem seems to be currently available^21^. A statistical method for model selection may in principle be able to find the number of communities, but it is at present too computationally demanding to be applicable to most but some small networks^13^. In our current study, we used two methods to determine the number of communities. First, we adopted the MDL as a yardstick to look for such network structures that can be encoded in minimum sizes. Second, for large networks, we devised a scheme of performing recursive bipartitioning until a terminal condition was met so that no number of communities needed to be determined *a priori*.

We presented two methods (i.e., NLC-EM and NLC-NMF) to learn the parameters of the model. The EM-based algorithm typically uses less memory but needs more time to converge than the NMF-based algorithm. While it is difficult to predict ahead of time which algorithm may provide better community results, the running time and memory requirement may be used as the criteria for choosing one of these algorithms for a given application. If the quality of resulting community structures is the main concern, we may run both algorithms and integrate their results following the ensemble learning paradigm (such as the consensus clustering method proposed by Lancichinetti & Fortunato^43^) to obtain a refined and better community structure, a direction we will take in our future work.

**Figure 10:**
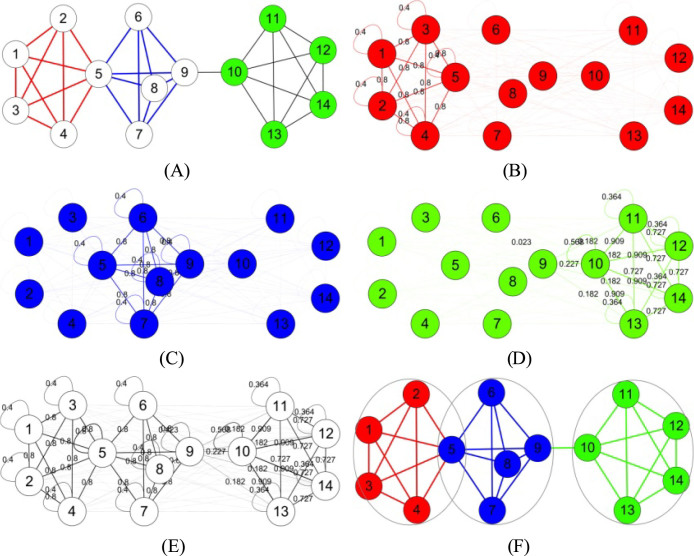
An illustration of NLC-EM for identifying hybrid node-link community structures. (A) A given network *G* with two link communities (in red and blue) and one node community (in green). (B), (C) and (D) The expected graph of the red, blue and green community. Note that the width of a link corresponds to its expected values, and the values smaller than 1.0e − 3 are omitted. (E) The expected graph of *G*, which is an ensemble of the expected graphs of the red, blue and green communities. (F) The inferred node and link communities colored in nodes or links respectively, and the final hybrid communities noted by three cycles.

Synthetic benchmarks have been designed for node communities^4^,^44^,^45^ and link communities^13^, separately. However, no suitable synthetic benchmark for hybrid node and link communities, such as that shown in Fig. 10(A), is current available. The designing of this type of benchmarks may be difficult because we not only need to give the node and link memberships, but also have to consider the final hybrid node-link community structure. We will also leave it to our future work.

## Methods

A key element of our method NLC is a probabilistic model to fit a given network. We are particularly interested in such a model that can accommodate both node and link communities. For clarity, we first describe the model and the algorithms for estimating its parameters. We then consider how to infer the hybrid node-link community structure from the model constructed.

### Stochastic model of node and link communities

#### The model

Our model consists of a set of probabilistic node and link communities that best fit a given network. In this model, a node (or a link) has a probabilistic membership in a node (or link) community, and the nodes (or links) that have high probabilities of a common membership form a probabilistic node (or link) community. In this formulation, we only need to focus on expected memberships. Specifically, given a network with *n* nodes, the model *G* can be specified by a set of parameters {*d_i_*_1_, *d_i_*_2_,…,*d_ic_*} for each node *i*, for *i* = 1,2,…,*n*, and a total of *c* communities, where *d_ik_* is proportional to the expected membership of node *i* in the *k*-th probabilistic community *G_k_*. That is, if *G_k_* is a node community, *d_ik_* is the expected node degree of *i* in *G_k_*, otherwise (i.e., *G_k_* is a link community), *d_ik_* is the expected number of links belonging to *G_k_* that node *i* connects to; *d_ik_*'s in both cases are equivalent. Then we will have 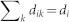 for each node *i*, where *d_i_* is the node degree of *i* in the given network.

It is critical to note that a community in the model has no further subdivision and can be regarded as a random graph with no community structure. Therefore, a random-graph null model (namely null model of modularity)^5^, which describes a random graph with a sequence of node degrees and with edges drawn at random among the nodes, can be adopted to characterize each of the communities. Following this null model, the expected number of links (or expected link weight) between nodes *i* and *j* in *G_k_* can be evaluated as
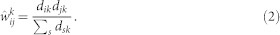
The expected number of links between nodes *i* and *j* in the given network can then be written as
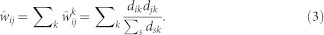
Note that multiple links between two nodes and self-edges are allowed here, which is typical for random graph models for simplicity^13^,^21^,^46^. The property of multiple links makes the model applicable to some weighted networks.

Intuitively, if node *i* is most likely to be a member of a given node community, it should have a high probability to connect with other nodes in that community, and consequently, nodes with a large proportion of membership to a common community tend to be densely connected to form the node community. Likewise, nodes with a large proportion of membership to a link community, as they have large numbers of adjacent links of a common type, tend to be highly connected through the same type of links to form the link community.

#### Parameter learning based on expectation-maximization algorithm

Our next step is to learn the parameters of the model to describe the community structure implied by *d_ik_*'s. Inspired by Ball's work^13^, this problem can be formulated by maximizing the likelihood of generating the given network from the model. Because the number of links between two nodes, *w_ij_*, is Poisson distributed with its expectation 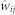
13,21, the probability for generating a graph *G* with adjacency matrix *A* = (*w_ij_*)*_n_*_ × *n*_ following (3) is
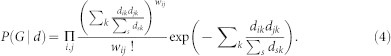


The best fit between the expected graph following (3) and the given network can be achieved by maximizing the likelihood function in (4). To be effective, the maximization is typically done with the logarithm of the likelihood, which has no effect on the position of the maximum. Applying logarithm to (4), rearranging, and dropping additive and multiplicative constants, we derive the log likelihood
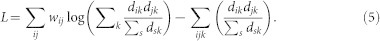


Since direct maximization of (5) is nontrivial, we adopt an expectation-maximization (EM) algorithm^47^. By applying Jensen's inequality to (5), we construct an auxiliary function,
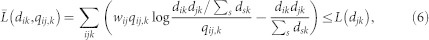
where the probabilities *q_ij,k_* can be freely chosen, provided that they satisfy 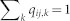. Thus 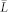 is a lowered bound of *L* and the equality holds at
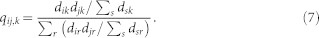


To maximize *L*, assume the current estimation of *d_ik_* to be 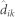. We have 
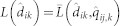
, where 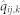 is derived from 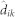 using (7). Then we keep 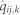 fixed in 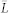 and maximize 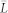 with respect to *d_ik_* under the constraints 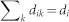. Introducing Lagrange multipliers *γ_i_* to incorporate these constraints, the Lagrange form of 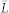 becomes
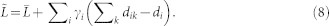
By taking partial derivative of 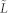 in (8), we obtain,
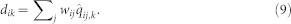
Therefore we have 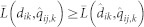. Now we can re-estimate the value of *q_ij,k_* using *d_ik_*, which leads to 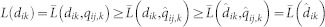. By alternating between the equations (7) and (9), the objective function *L* monotonically converges to a local minimum of the log-likelihood function.

#### Parameter learning based on nonnegative matrix factorization

Here we introduce an alternative method to learn the parameters of the model. We use a squared loss, instead of a Poisson distribution, to derive their objective functions. Then the problem of fitting the model to the data of *G* can be cast as the following optimization problem,
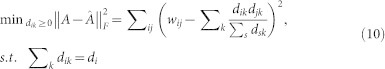
where ||.||*_F_* denotes the Frobenius norm. The best fit between the expected graph with adjacency 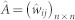 and a given network *G* with adjacency *A* = (*w_ij_*)*_n_*_ × *n*_ can be achieved by optimizing (10). We use a nonnegative matrix factorization (NMF) method to solve the optimization in (10), as described as follows.

We first introduce an auxiliary matrix *X*, where *X_ik_* is defined as
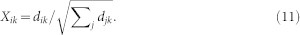
The loss function in (10) can be rewritten as a constrained NMF problem,
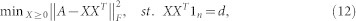
where *d* = (*d*_1_, *d*_2_,…, *d_n_*)^T^. It is nontrivial to directly optimize (12) with the hard constraints. We relax this optimization problem by introducing a penalty term that represents the hard constraints into the objective function, arriving at minimizing the following objective function,
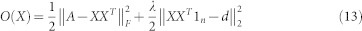
where λ is a hyperparameter that reflects the importance of the hard constraints. Violation to more hard constraints incurs a higher penalty to the objective function. In our experiments, we first get an initial value of *X*_0_ by setting λ = 0. We then restart the optimization with *X* *=* *X*_0_ and set λ to a relatively large number, e.g., 1000, to minimize the chance of violating the degree constraints. The purpose of the initialization is to restrict the search for a model to start from some good approximations. Similar to other forms of NMF, the objective function in (13) is not convex w.r.t. *X*, so that it is computationally intractable to find global minima. Therefore, the gradient descent method is adopted to search for local minima, which can be implemented in a multiplicative updating algorithm similar to the method for SNMF^48^. In order to derive the updating rule, a Lagrange multiplier matrix *Θ* for the nonnegative constraints on *X* is introduced to (13), resulting in the following equivalent objective function,
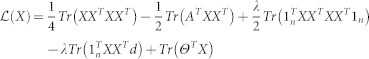
For any stationary state, we have
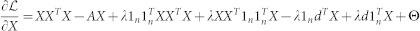
Using complementary slackness condition (Θ)*_ik_*(*X*)*_ik_* *= * 0, we have the following equation,
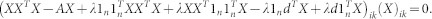
This leads to the following update rule for *X*:
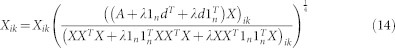
When the update rule converges, shown in Theorem 1 below, the solution satisfies the Karush-Kuhn-Tucker (KKT) conditions^49^.

#### Theorem 1

Function *O* in (13) is non-increasing under the updating rule in (14). *O* is invariant under these updates if and only if *X* becomes stationary (see Supplementary Information for proof).

Finally, when the method converges, the parameters *d_ik_* of the model can be computed using (11) as follows,
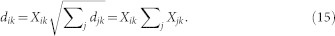


### Inferring hybrid community structure

Even with a model of node and link communities constructed for a given network, it is not straightforward to infer community structures. This entails inferring the nodes or links, respectively, in a node or link community, and determining the type (i.e., node or link) of each of the communities. For clarity and efficiency, we consider these two issues separately.

#### Inferring community structure given the types of communities

Determining the structure of a community amounts to determining its members. Assume that the type of each of the communities is known. We first define two sets of variables: *S_i_^k^* represents the probability or probabilistic membership that node *i* belongs to the *k*-th community *G_k_*, and *R_ij_^k^* denotes the probability that a link <*i*, *j*> belongs to *G_k_*. Then, *S_i_^k^* can be evaluated as
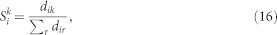
and *R_ij_^k^* can be written as
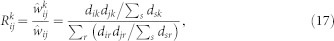


The probabilistic memberships of communities are used to infer deterministic memberships of communities, thus forming deterministic communities defined as {*H*_1_, *H*_2_,…,*H_c_*}. If *H_k_* is a node community, it will consist of all nodes *i* satisfying *arg*max*_r_*{*S_i_^r^* | *r* = 1,2,…,*c*} = *k*; if *H_k_* is a link community, it will contain all links <*i*, *j*> satisfying *arg*max*_r_*{*R_ij_^r^*| *r* = 1,2,…,*c*} = *k*.

#### Determining the types of communities

Determining the type of each of the communities separately seems to be nontrivial, and may not necessarily give rise to a global optimality for the whole network either. Here we adopt a global method for this problem. Since there are *c* communities, each of which can be either a node or link community, there are a total of 2*^c^* possible combinations of hybrid node-link communities. In order to identify the best among these combinations, we need a quality metric to measure the quality of a candidate combination of communities.

The map equation for overlapping communities^16^ measures how well we can compress a description of flow in the network when it is partitioned into communities with possible overlaps. The idea follows the principle of Minimum Description Length (MDL) that any regularity in the data can be used to compress the data^17^. If one can find a way to encode the path of a random walk on the network and consider the overlapping community structure as the regularity (that a random walker is statistically likely to spend long periods of time within certain clusters of nodes), the description length of the path can be used to evaluate the quality of the overlapping communities. In the map equation, the encoding rule for the path description can be described as follows. It uses the codebook at two levels: the first level code describes the communities with overlaps and the second level code distinguishes a specific node from others in the same community. In this strategy, a community code (first level) should be recorded in the path description when the random walk enters a new community, and the random walk inside the community can be uniquely described by only recording the second level code. Besides, an exit code should be assigned to each community and it should be recorded when the random walk exits the community, so that the first level code and the second level code can be distinguished (see Supplementary Information for detail).

The map equation measures how well we can compress a path description in the network when considering the significance of community structure, thus it can be used to determine which partitioning scheme – node community, link community or hybrid community – can subtract more unknown information and better represent the organization structure of the network. Therefore, we adopted the map equation^16^ here to determine the type of communities. For clarity, we use *V_k_* or *E_k_* to explicitly indicate that the *k*-th deterministic community *H_k_* is a node or link community, respectively. Assume that *H* = {*H_k_* | *k* = 1,2,…,*c*} is a candidate hybrid node-link community structure, where *H_k_* is either *V_k_* or *E_k_*. Let *L*(*H*) be the value of MDL for *H*. A straightforward way to finding the best hybrid node-link community structure is to enumerate all possible combinations for *H* to find the one with the minimum value of MDL. This exhaustive search may become computationally expensive for large networks. Here we offer an alternative, an effective heuristic, to this search problem, which takes the following steps.

**S1** Initialize a candidate hybrid community structure *H*: for community *k*, randomly assign either *V_k_* or *E_k_* to *H_k_*;

**S2** Update *H*: for community *k*, swap the current *H_k_* to the other community (*V_k_* or *E_k_*) if doing so reduces *L*(*H*);

**S3** Repeat S2 until *L*(*H*) cannot be reduced further, or the maximal number of iterations has been executed.

### A working example of NLC

Here we illustrate the procedure of our NLC method with an example. For simplicity, we present the results of NLC-EM here. The observed network is shown in Fig. 10A. Given the model parameters *d_ik_* (see Table 3 and discussion below), we can form the expected graphs of all the communities of the observed network (Fig. 10B, 10C and 10D) according to (2). Further, we can form the expected graph of the whole network *G* (Fig. 10E) according to (3), which is an ensemble of the expected graphs of all its communities. However, since the model parameters are unknown *a priori*, we fit network and its expected graph by optimizing (4) to learn the best *d_ik_* (Table 3). Subsequently, we infer all the node and link communities according to (16) and (17), and identify the final network community structure (Fig. 10F) based on the principle of minimum description length.

As shown in Fig. 10(F), our NLC method can not only infer the node and link communities (colored on nodes and links, respectively), but also derive the hybrid node-link community structure (noted by three cycles) which faithfully corresponds to the ground-truth. In comparison, the node community detection methods compared^6^,^21^ only inferred node partitions, corresponding to colors on nodes in Fig. 10(F); the link community detection methods^10^,^13^ only inferred link partitions, corresponding to colors on links in Fig. 10(F); and both of them cannot perfectly classify the network. This may further validate the flexibility and effectiveness of our hybrid node and link community scheme compared with single type of schemes.

## Author Contributions

D.H. and W.Z. designed the study; D.H., D.J. and Z.C. performed the experiments, analyzed the data and prepared the figures; D.H., D.J. and W.Z. wrote the paper. All authors reviewed the manuscript.

## Supplementary Material

Supplementary Information Supplementary Information: Identification of hybrid node and link communities in complex networks

## Figures and Tables

**Table 1:** Comparison of the MDLs from the new NLC method (including NLC-EM and NLC-NMF) and 5 existing algorithms on 9 real-world networks obtained from Newman's website^50^. Here, *n* is the number of nodes and *m* the number of links, and ‘node', ‘link', ‘overlap', and ‘hybrid' denote node, link, overlapping, and hybrid communities, respectively. The mean values and standard deviations are based on 50 runs, and Louvain, LC and CPM are deterministic algorithms without standard deviations. The shorter the MDL of an overall community structure, the better the structure is. The best MDLs for these networks are in bold and underlined

			Methods						
Datasets/MDLs	*n*	*m*	Louvain (node)	LC (link)	CPM (overlap)	NModel (node)	LModel (link)	NLC-EM (hybrid)	NLC-NMF (hybrid)
Zachary's karate club	34	78	4.3359	5.2502	5.8552	5.2288 ± 0.0211	4.2966 ± 0	4.2966 ± 0	**4.2611 ± 0**
American college football	115	613	5.4982	7.7749	5.5376	5.7654 ± 0.0640	6.1494 ± 0.0345	5.4487 ± 0	**5.4466 ± 0**
Les Miserables	77	254	4.7632	5.4371	5.4126	5.2338 ± 0.0659	4.7784 ± 0.0270	**4.6809 ± 0.0106**	4.7665 ± 0.0096
Dolphin social network	62	160	4.8859	6.6531	6.0031	5.4426 ± 0.1419	4.9681 ± 0.0397	**4.8389 ± 0.0121**	4.8505 ± 0
High school friendship	69	220	4.7981	6.1582	5.8842	4.9198 ± 0.0572	4.8204 ± 0.0412	4.7575 ± 0.0033	**4.7368 ± 0**
Political books	105	441	5.5836	7.6100	5.7855	5.5648 ± 0	5.5796 ± 0.0299	**5.5413 ± 0.0001**	5.5634 ± 0
Jazz musicians collaborations	198	2,742	6.8745	8.9557	7.3312	6.8675 ± 0	6.8742 ± 0.0013	**6.8529 ± 0**	6.8639 ± 0
C. Elegans neural network	297	2,148	7.6309	11.2642	8.0112	7.9074 ± 0	7.5873 ± 0.0116	**7.5631 ± 0.0008**	7.5818 ± 0.0028
E-mail network URV	1,133	5,451	8.5428	12.2934	9.5409	8.5181 ± 0.0558	8.9433 ± 0.0354	**8.4762 ± 0.0299**	8.5353 ± 0.0518

**Table 2:** Comparison of the NMIs accuracy of different methods on 6 Stanford large networks with ground-truth of overlapping communities^22^. Here, *n* is the number of nodes, *m* the number of links and *c* the number of communities. M denotes a million and k one thousand. The mean values and standard deviations are based on 50 runs, and Louvain, LC and CPM are deterministic algorithms without standard deviations. The larger the NMI of an overall community structure, the better the structure is. The best NMIs for these networks are shown in bold and underlined

				Methods						
Datasets/NMIs (%)	*n*	*m*	*c*	FUA (node)	LC (link)	CPM (overlap)	NModel (node)	LModel (link)	NLC-EM (hybrid)	NLC-NMF (hybrid)
LiveJournal	4.0M	34.9M	310k	20.07	14.77	18.84	27.64 ± 0.56	23.69 ± 0.48	28.74 ± 0.49	**41.02 ± 1.15**
Friendster	120M	2,600M	1.5M	28.65	17.18	27.59	32.82 ± 1.07	32.36 ± 0.57	**38.97 ± 0.51**	23.50 ± 0.62
Orkut	3.1M	120M	8.5M	25.60	17.73	26.54	26.90 ± 0.55	23.69 ± 0.43	28.59 ± 0.40	**33.83 ± 0.24**
Youtube	1.1M	3.0M	30k	24.06	17.81	13.80	17.82 ± 0.60	29.91 ± 0.60	**33.92 ± 0.46**	31.70 ± 0.14
DBLP	0.43M	1.3M	2.5k	16.83	14.12	17.99	15.20 ± 0.51	13.71 ± 0.52	14.98 ± 0.30	**35.49 ± 0.36**
Amazon	0.34M	0.93M	49k	24.73	17.56	18.10	26.70 ± 0.40	24.74 ± 0.70	29.24 ± 0.59	**41.44 ± 0.62**

**Table 3:** The model parameters *d_ik_*'s learned by EM algorithm

*d_ik_*	*i* = 1	*i* = 2	*i* = 3	*i* = 4	*i* = 5	*i* = 6	*i* = 7	*i* = 8	*i* = 9	*i* = 10	*i* = 11	*i* = 12	*i* = 13	*i* = 14
*k* = 1	4.73e − 124	3.04e − 123	1.92e − 123	1.02e − 123	2.84e − 17	3.89e − 15	4.30e − 15	3.84e − 15	0.999991	4.999991	4	4	4	4
*k* = 2	3.999987	3.999987	3.999986	3.999987	3.999946	3.24e − 20	5.36e − 19	8.14e − 20	1.15e − 14	4.14e − 103	0	0	0	0
*k* = 3	1.35e − 05	1.35e − 05	1.35e − 05	1.35e − 05	4.000054	4	4	4	4.000009	9.04e − 06	6.50e − 87	8.66e − 86	8.05e − 86	4.21e − 86
