# Supplementary material for: Identification of hybrid node and link communities in complex networks
Source: Sci Rep. 2015 Mar 2;5:8638. doi: 10.1038/srep08638 (PMC4345336; doi:10.1038/srep08638)
Supplement: Supplementary Information — Identification of hybrid node and link communities in complex networks [file srep08638-s1.pdf]

Supplementary Information:
Identification of hybrid node and link communities in complex networks

Dongxiao He, Di Jin, Zheng Chen & Weixiong Zhang

I The map equation for overlapping communities

Here we provide the detail of the map equation for overlapping communities[1,2]. Define a cover M of a network N as a set of communities such that each node is assigned to at least one community. The map equation $L(M)$ gives the average number of bits per step that it takes to describe an infinite random walk on the network with cover M:

$$L(M) = q_{out} H(Q) + \sum_{k=1}^{c} p_{in}^{k} H(P^{k}) , \tag{1}$$

where k is the index of community (note that we also use k denote the k-th community for simplification), i the index of node, and c the number of communities; $q_{out} = \sum_{k=1}^{c} q_{out}^{k}$ is the total probability of using the first level codebook where q_{out}^{k} is the probability of using the first level code for community k; $p_{in}^{k} = q_{out}^{k} + \sum_{i \in k} p_{i}^{k}$ is the probability of using the second level codebook and the exit code for community k, and p_i^k is the probability of node i being visited as a number of community k, which is equal to the probability of using the second level code for node i in community k. $H(Q)$ is the average description length of the first level codebook:

$$H(Q) = -\sum_{k=1}^{c} \left(\frac{q_{out}^{k}}{q_{out}} \log \frac{q_{out}^{k}}{q_{out}} \right), \tag{2}$$

while $H(P^k)$ is the description length of the second level codebook for community k:

$$H(P^{k}) = -\frac{q_{out}^{k}}{p_{in}^{k}} \log \frac{q_{out}^{k}}{p_{in}^{k}} - \sum_{i \in k} \left(\frac{p_{i}^{k}}{p_{in}^{k}} \log \frac{p_{i}^{k}}{p_{in}^{k}} \right). \tag{3}$$

In order to compute the map equation for overlapping communities, we need to calculate the visit rates p_i^k for all communities $k \in M_i$ which a node i is assigned to, and the exit probabilities q_{out}^{k} of all communities. Here M_i denotes the set of community indexes of some node i.

The switches between multiply assigned nodes and overlapping communities are straightforward. Whenever the random walk arrives at a node that is assigned to multiple communities, it remains in the same community if possible or switches to one of the other communities randomly otherwise. For example, assuming that the walk is at a node j in community s, it remains in community s when moving to node i if node i is assigned to community s, $s \in M_i$. But if node i is not assigned to community s, $s \notin M_i$, it switches with equal probability $1/|M_i|$ to any of the communities to which node i is assigned. If the transition

function is defined as

$$\delta_{js\to ik}=\begin{cases}1 & if\ s=k\\ \frac{1}{|M_i|} & if\ s\neq k\quad and\quad s\notin M_i\\ 0 & if\ s\neq k\quad and\quad s\in M_i\end{cases},\tag{4}$$

the visit rates can then be written as

$$p_i^k=\sum_j\sum_{s\in M_j}\left(p_j^s u_{ji}\delta_{js\to ik}\right),\tag{5}$$

where $u_{ji}=w_{ji}/\sum_r w_{jr}$ denotes the probability of the random walk moving from nodes j to i. The visit rates p_i^k can be computed with the fast iterative algorithm BICGSTAB[3]. Then since every node in community k guides a fraction $\sum_{j\notin k}u_{ij}$ of its conditional probability p_i^k to nodes outside community k, the exit probability of community k is

$$q_{out}^k=\sum_{i\in k}\left(p_i^k\sum_{j\notin k}u_{ij}\right).\tag{6}$$

Using (5) and (6), the map equation in (1) can be derived.

II The proof of Theorem 1

We adopt the auxiliary function approach used in Expectation-Maximization and Nonnegative Matrix Factorization. The basic idea is to construct an auxiliary function $C(X,\tilde{X})$ such that:

$$O(X)=C(X,X)\leq C(X,\tilde{X})\leq C(\tilde{X},\tilde{X})=O(\tilde{X})$$

If we can minimize $C(X,\tilde{X})$ w.r.t to X, we are guaranteed to drive $O(X)$. Note that,

$$O(X)=\frac{1}{4}Tr(XX^TXX^T)-\frac{1}{2}Tr(A^TXX^T)+\frac{\lambda}{2}Tr(1_n^TXX^TXX^T1_n)-\lambda Tr(1_n^TXX^Td)$$

$$\leq\frac{1}{4}Tr(P\tilde{X}\tilde{X}^T)+\frac{\lambda}{2}Tr(P1_n1_n^T\tilde{X}\tilde{X}^T)-\frac{1}{2}Tr(A^TXX^T)-\lambda Tr(d1_n^TXX^T)$$ (by Lemma 6 of [4])

$$\leq\frac{1}{4}Tr(R^T\tilde{X}\tilde{X}^T\tilde{X})+\frac{\lambda}{4}Tr(R^T1_n1_n^T\tilde{X}\tilde{X}^T\tilde{X})+\frac{\lambda}{4}Tr(\tilde{X}^T1_n1_n^T\tilde{X}\tilde{X}^TR)-Tr(Z^TA\tilde{X})-$$

$$\lambda Tr(Z^Td1_n^T\tilde{X})-\lambda Tr(\tilde{X}^Td1_n^TZ)-\frac{1}{2}Tr(\tilde{X}^TA\tilde{X})-\lambda Tr(\tilde{X}^Td1_n^T\tilde{X})$$ (by Lemma 7 and 3 of [4])

$$\equiv C(X,\tilde{X}),$$

where $P_{kl}=[XX^T]_{kl}^2/[\tilde{X}\tilde{X}^T]_{kl}$, $R_{ik}=[X]_{ik}^4/[\tilde{X}]_{ik}^3$, and $Z_{ij}=\tilde{X}_{ij}\ln(X_{ij}/\tilde{X}_{ij})$. The equality clearly holds when $X=\tilde{X}$. Then $C(X,\tilde{X})$ satisfies the conditions of being an auxiliary function for $O(X)$. We can define the series of updating rules as:

$$X^{(t+1)}=\min_X C(X,X^{(t)})$$

$$\frac{\partial C(X,\tilde{X})}{\partial X_{ik}}=\frac{X_{ik}^3}{\tilde{X}_{ik}^3}[\tilde{X}\tilde{X}^T\tilde{X}+\lambda 1_n1_n^T\tilde{X}\tilde{X}^T\tilde{X}+\lambda\tilde{X}\tilde{X}^T1_n1_n^T\tilde{X}]_{ik}$$

$$-\frac{\tilde{X}_{ik}}{X_{ik}}[A\tilde{X}+\lambda d 1_n^T\tilde{X}+\lambda 1_n d^T\tilde{X}]_{ik}=0$$

So we have the update rule for X as in Eq. (14) in the main text.

References

1. Esquivel, A. V. & Rosvall, M. Compression of flow can reveal overlapping-module organization in networks. *Phys. Rev. X* **1**, 021025; DOI:10.1103/PhysRevX.1.021025 (2011).
2. Grünwald, P. D. *The Minimum Description Length Principle*. (Cambridge, MA, USA: The MIT Press, 2007).
3. van der Vorst, H. A. BI-CGSTAB: a fast and smoothly converging variant of BI-CG for the solution of nonsymmetric linear systems. *SIAM J. Sci. Comput.* **13**, 631-644; DOI:10.1137/0913035 (1992).
4. Wang, F., Li, T., Wang, X., Zhu, S. & Ding, C. H. Q. Community discovery using nonnegative matrix factorization. *Data Min. Knowl. Discov.* **22**, 493-521; DOI:10.1007/s10618-010-0181-y (2011).
